# A novel surgical technique for cervical laminoplasty in patients with multilevel cervical spondylotic myelopathy: A case report and literature review

**DOI:** 10.3389/fsurg.2023.1078138

**Published:** 2023-03-03

**Authors:** Xinyi Huang, Daming Liu, Yipeng Yang, Haiyang Qiu, Zhensheng Ma, Wei Lei, Yang Zhang

**Affiliations:** Department of Orthopedics, Xijing Hospital, The Air Force Medical University, Xi’an, China

**Keywords:** cervical laminoplasty, multilevel spondylotic myelopathy, alternating side, axial neck pain, novel surgical technique

## Abstract

Cervical laminoplasty is a posterior-based surgical decompression technique for the treatment of multilevel cervical spondylotic myelopathy (CSM) that may improve the preservation of cervical mobility, spinal canal structure, and natural lordosis. Although this procedure is considered to be comparatively safe, with fewer complications than those seen with laminectomy, several postoperative problems have been noted, including axial neck pain, C5 nerve palsy, and failed resolution of radiculopathy. Hence, various modifications have been made to improve the safety and effectiveness of this technique. Here, we report the case of a 74-year-old man with multilevel CSM who underwent posterior cervical laminoplasty in the C3–C7 segments using a novel surgical technique, termed alternating-side cervical laminoplasty. Preoperative and postoperative assessments, including visual analog scale, modified Japanese Orthopaedic Association, neck disability index scores, and imaging data, were collected and analyzed. The results of a 5-year follow-up indicated that the patient recovered well, with no development of axial neck pain. This is the first report of this modified open-door laminoplasty, which we propose may be a better surgical option for preventing postoperative axial neck pain in patients with multilevel CSM. Additionally, opening the laminae on the alternating sides during laminoplasty could provide a flexible approach to complete decompression on different radiculopathy sides.

## 1. Introduction

Cervical laminoplasty is a posterior technique that can be performed to achieve multilevel posterior decompression of the spinal canal while maintaining alignment and mobility of the spine (1). Initially, this technique was suggested for patients with cervical spondylotic myelopathy (CSM) resulting from multilevel stenosis secondary to ossification of the posterior longitudinal ligament (OPLL). However, it is currently also used for multiple herniated cervical discs accompanying spinal stenosis and multilevel spondylosis-associated spinal cord injury (2, 3). Indeed, cervical laminoplasty was developed as an alternative to laminectomy with the aim of avoiding the original complications of laminectomy alone (4), such as postoperative segmental instability, recurrence of spinal cord compression, kyphosis, perinerve adhesion, late neurological deterioration, and so on (5).

Based on laminectomy, the first laminoplasty technique termed Z-plasty was introduced by (6, 8). This technique involved removal of the spinous process, thinning of the laminae in which the z-shaped cuts were made next, followed by elevation and fixation with sutures to reconstruct the expanded spinal canal. Unfortunately, owing to its complicated procedure, Z-plasty was not widely available. After the 1970s, Hirabayashi et al. reported the open-door laminoplasty, while Kurokawa et al. developed the double-door laminoplasty technique (also called French-door laminoplasty or spinous process-splitting laminoplasty) (9, 10). The former involved excision of the lamina border on one side and drilling of the bony gutter on the other side so that the lamina would be pushed laterally as if to open a door, while the latter involved opening the spinal canal in the midline bilaterally (like a French-door) by splitting the spinous process. Since these two prototype techniques were original published, various modifications of cervical laminoplasty have been developed with the aim of improving the safety and effectiveness of the procedure (Table 1). For example, Hirabayashi et al. secured the laminae to the facet by using sutures, while O'Brien et al. used titanium miniplates for security (11). In addition, in the Tomita and Morimoto modifications (12, 13), bone graft was used as a spacer in the final step of the French-door laminoplasty, including later ceramic laminas and hydroxyapatite spacers (14–16). More recently, several clinical studies have focused on preserving muscle attachment to enable dynamic stabilization of the cervical spine by the neck extensor muscles (17–20).

**Table 1:** Development of the surgical technique of cervical laminoplasty.

Type	Representative	Technical feature	Advantages of modification
Lamina-Z-plasty	Oyama et al. (1973) (8)	Z-shaped cuts made in each lamina and fixed with sutures	Retains support; prevents “laminectomy membrane” formation
Open-door laminoplasty	Hirabayashi et al. (1978) (9)	Elevates the laminae on the hinge, secured to the facet using sutures	Operative technique is relatively easy and safe
Double-door laminoplasty	Kurokawa et al. (1982) (8)	Spinous processes are split in the midline and are maintained open	Achieves symmetrical expansion of the spinal canal
Hardware-augmented	Hase et al. (1991) (15)	Uses ceramic laminas	Provides a simpler, safer, and more effective method of fixation
	Nakano et al. (1992) (16)	Uses a hydroxyapatite spinous process spacer	
	O'Brien et al. (1996) (11)	Uses titanium miniplates to secure the elevated laminae	
Muscle-sparing	Shiraishi (2002) (34)	Preserves the attachments of the semispinalis and multifidus muscles to the spinous processes	Reduces postoperative axial pain
	Takeuchi et al. (2005) (20)	Preserves the semispinalis cervicis insertion into C2	
	Hosono et al. (2006) (19)	Preserves the C7 spinous process and the origin of the trapezius and rhomboideus minor muscles	
Instrumented	Lee et al. (2016) (23)	Insertion of translaminar screws, fixed in the contralateral lateral mass	Maintains spinal stability and prevents postoperative axial pain and deformity
	Nasto et al. (2017) (22)	Uses trapezoidal maxillofacial titanium miniplates with bone graft for fixation	Safe, reproducible, and alternative technique

Although considerable progress has been made in the last few decades, some challenges induced by laminoplasty—such as kyphosis, axial neck pain, and C5 nerve palsy, which can have a significant impact on patients’ quality of life—are yet to be solved (21). In order to optimize these postoperative residual problems, instrumented techniques are constantly being innovated with the invention of new internal fixation devices (22, 23). Nevertheless, few reports have focused on modifications of the original technique. In the present study, we review the case of one patient who underwent a novel surgical cervical laminoplasty to evaluate whether this modification resulted in any beneficial effects, and further provide a review of literature to discuss modifications to the technique.

## 2. Case presentation

### 2.1. Patient characteristics

We present the case of a patient who underwent cervical laminoplasty in the C3–C7 segments, performed in 2017. The 74-year-old man was admitted to our center with complaints of loss of strength and persistent numbness in his upper limbs for 2 years. Additionally, he experienced progressive walking disturbance in both legs for 1 year.

**Figure 1:**
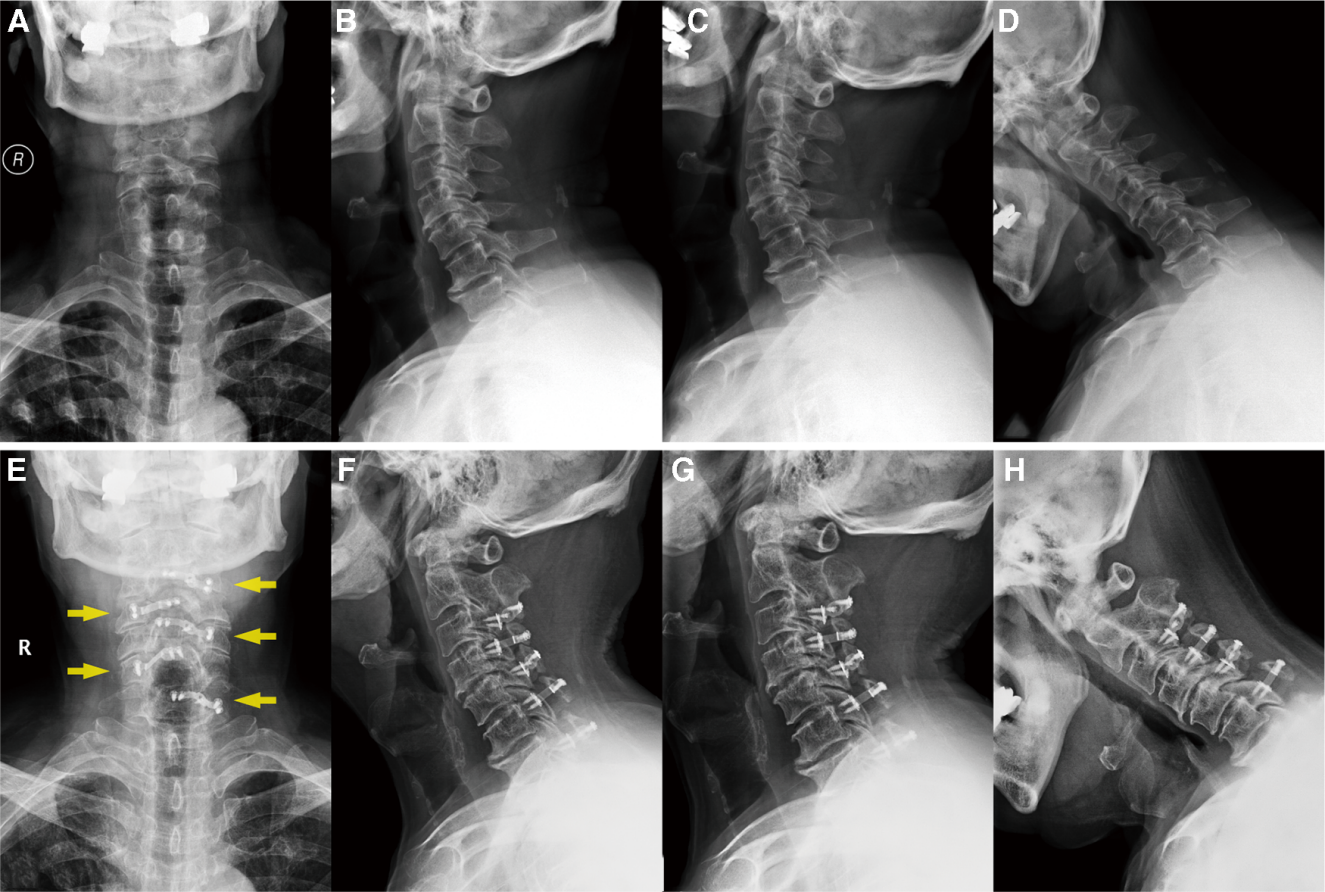
(**A–D**) Preoperative x-rays of the cervical spine (anteroposterior, lateral, hyperextension, and flexion radiographs, respectively), showing that the vertebral physiological curvature straightened with a certain degree of degenerative change. (**E–H**) Postoperative x-ray after 5 years, demonstrating the position of the internal fixation plates (yellow arrows) and that the cervical curvature and the range of motion have remained the same.

**Figure 2:**
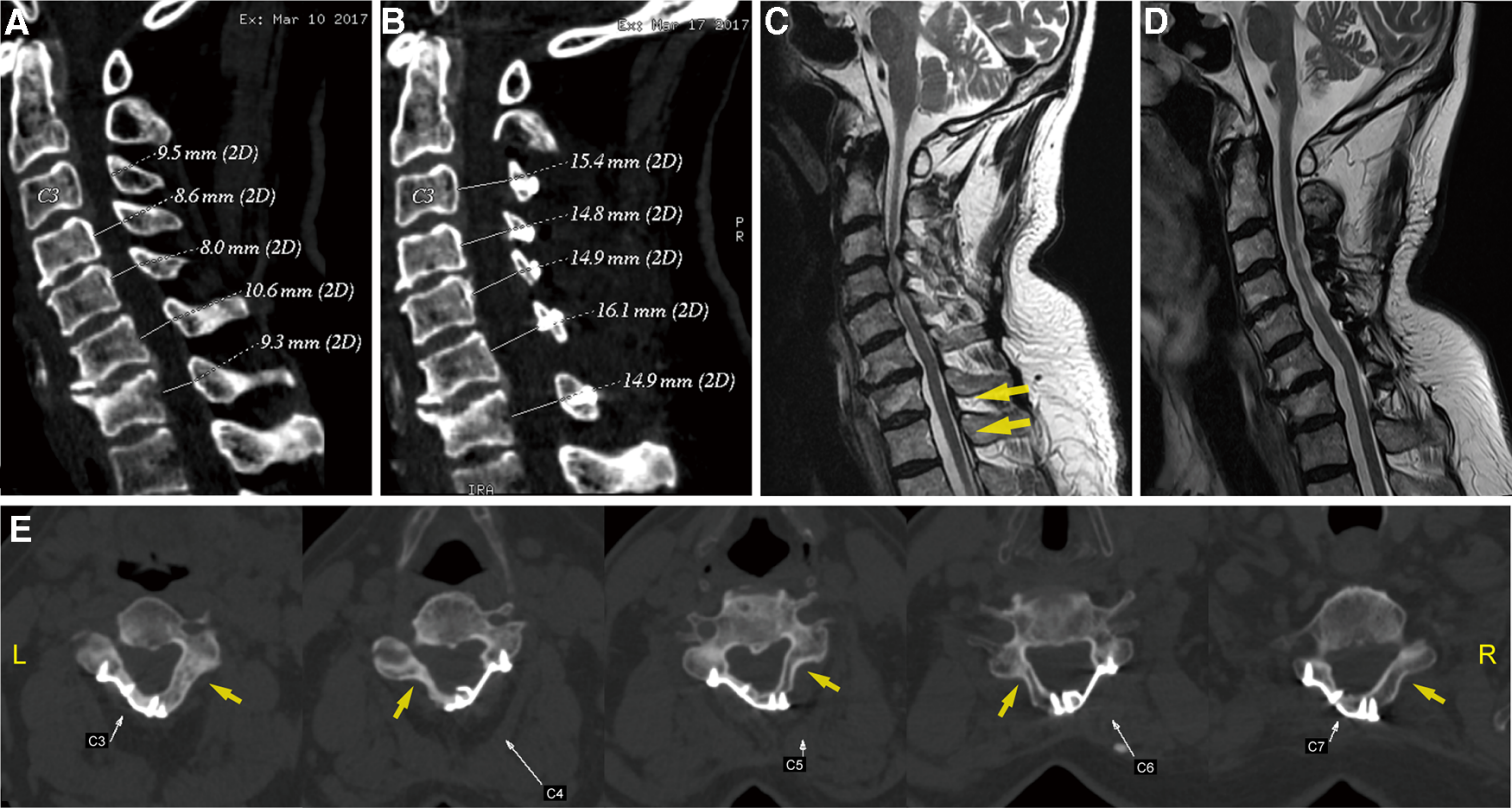
(**A,B**) Preoperative and postoperative sagittal CT scans, illustrating the changes in the cervical spinal canal diameter. Sagittal T2-weighted MR images: (**C**) preoperative scan, showing a cervical stenosis and varying degrees of disc herniation at the C3–C7 levels, with particular severeness at C3–C4 and C4–C5 (yellow arrows); (**D**) 5-year postoperative scan, showing adequate spinal cord decompression. (**E**) Axial CT scan of the operated segments 5 years after the operation, suggesting a large fusion on the laminar hinge position (yellow arrows) and that the laminar doors have been kept open by the plates. CT, computed tomography; MR, magnetic resonance.

Upon admission, the patient’s general condition was normal, except for a visual analog scale (VAS) of neck pain score of 3, modified Japanese Orthopaedic Association (mJOA) score of 8, and neck disability index (NDI) score of 26, indicating severe dysfunction. Neurological examination revealed right-side dominant weakness (i.e., power as evaluated by manual muscle testing: 4/5 for the deltoid muscles, biceps, and triceps of the right upper limb, and the same for the wrist flexors and wrist extensor, except for the finger flexor, the finger extensor, and the intrinsic muscles of the hand, which were normal), hypesthesia of the radial side of the right forearm and right thumb, and a right positive Hoffmann’s sign. Bilateral hyperreflexia of the patellar reflex, right hyperreflexia of the ankle reflex, and a positive Babinski test result were also observed. Radiographic examination, including anteroposterior, lateral, hyperextension, and flexion radiographs of the cervical spine, showed that the height of intervertebral spaces was decreased in the C3–C7 segments, notably at the C4–C5 and C6–C7 levels. In addition, with degenerative changes at the edges of the vertebrae and straightening of the physiological curvature, the cervical spine range of motion was diminished, but remained relatively stable (Figures 1A–D). Sagittal computed tomography (CT) of the cervical spine further showed osteophytes formation at the anterior edges of the vertebrae of C4–C7 and multilevel spinal canal stenosis (Figure 2A). Furthermore, magnetic resonance imaging (MRI) demonstrated cervical stenosis at the C3–C7 levels with varying degrees of disc herniation and compression of corresponding dural sacs and spinal cord, particularly severe at the C3–C4 and C4–C5 levels (Figure 2B). CSM was diagnosed based on the patient’s clinical presentation and imaging findings. Posterior cervical laminoplasty (PCL) at the C3–C7 levels was deemed necessary to decompress the spinal canal.

Considering the safety and stability of the present surgical techniques, open-door laminoplasty and fixation with plates is generally preferred in our hospital. During the preoperative conversation, the patient highlighted the need to minimize postoperative neck pain as much as possible, as this had already considerably reduced his quality of life. However, in this unilateral open-door laminoplasty, an asymmetric expansion of the canal is created, resulting in skeletal and muscular asymmetry, which may further lead to postoperative axial pain (24, 25). In light of this problem, our team has designed a novel surgical technique in which the laminar door is opened alternatively to conserve the posterior structure of cervical spine symmetry in an attempt to reduce axial pain. A finite element (FE) analysis has been performed in advance, confirming that this technique preserves the symmetry of the cervical structure, thereby promoting improved balance during right and left lateral flexion and rotation.

Given the patient’s needs, we believed that this surgery was suitable and that it may be able to achieve satisfactory results. As such, the patient and his family were fully informed of the risks and benefits of the procedure, and informed consent was obtained. The patient underwent surgery in March 2017.

### 2.2. Surgical procedure

The patient was placed in a prone and reverse Trendelenburg position. His head and neck were kept in slight flexion using a Mayfield head holder, with the sagittal line of the neck parallel to the floor. The bony landmarks were palpated to determine the level of the C7 spinous processes. First, a midline posterior approach focusing on C2–C7 was performed, and the laminae were exposed to the midportion of the lateral masses so that the muscle origins over the lateral half could be preserved. The extensor muscles were subsequently detached from the lower laminar margin of C2 to allow access to the C2–C3 interlaminar space. Second, the spinous processes of C3–C7 as well as part of the lower laminar margin of C2 and the upper laminar margin of C7 were removed using a Kerrison rongeur. Bone wax was used to achieve hemostasis on the bone surfaces. Third, the junctions between the lateral parts of the lamina and the lateral mass were identified at each level where the side troughs were prepared. The junctions were thinned using a high-speed drill until the dorsal cortex was removed, forming hinge side troughs, which yielded slightly with a moderate bending force. On the contralateral side, the junctions were excised to construct the open side troughs, and the ligamentum flavum, facet capsules, and veins were carefully divided, as required. It is worth noting that this procedure requires transverse excisions of the ligamentum flavum in each laminar space from C2 to T1 to allow the adjacent laminae to be independent. After the adhesions have been separated from the dura by the use of a nerve hook, the laminae are gradually opened by applying a slight opening force.

In contrast to the traditional open-door laminoplasty, which involves opening of the unilateral side of the spinal canal, we designed this technique to open the laminae on the alternating side. Thus, the hinge side trough was made on the right and the open side trough on the left at the C3, C5, and C7 segments, while the hinge side trough was made on the left and the open side trough on the right at the C4 and C6 segments, so that the laminar doors opened alternatively from C3 to C7. Subsequently, all the laminae were lifted, and an appropriately sized laminoplasty plate was selected for each level using a bone trial. Then, self-tapping screws were inserted using a self-holding screwdriver to anchor the centerpiece plates (SOFAMOR DANEK, Medtronic, Memphis, TN, United States) to the lateral mass and lamina at each segment for stabilization and support. Finally, we checked whether the decompression was sufficient and observed the placement of the plate *via* bedside radiography, and adjustments were made until a satisfactory result was obtained. A deep drain was placed and the wound was closed.

### 2.3. Postoperative information

**Figure 3:**
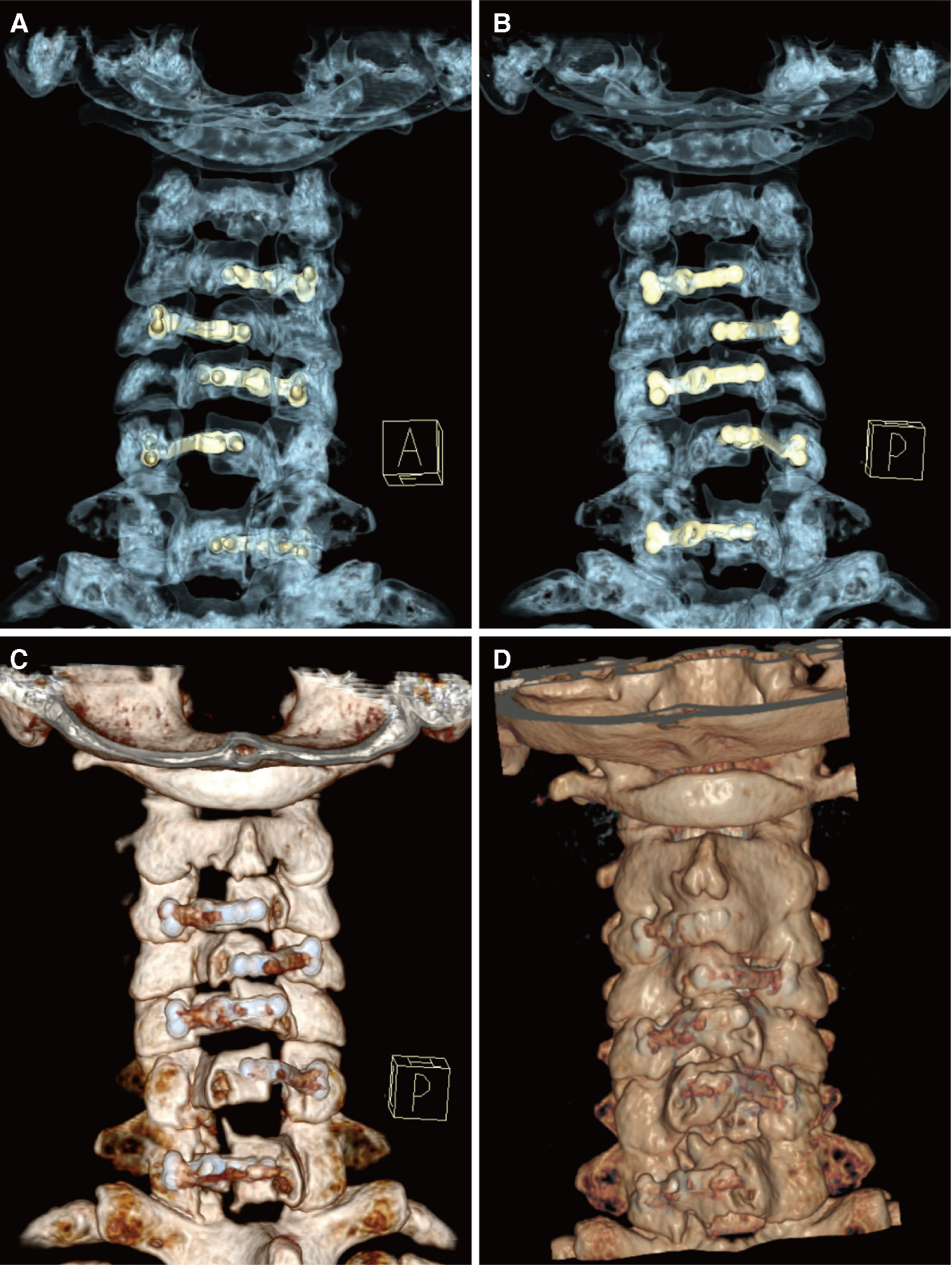
(**A,B**) Three-dimensional reconstructions of CT images (**A**: anterior view, **B**: posterior view), to allow clear visualization of the operating feature of the internal fixation plates during this novel technique. (**C,D**) Posterior views of the reconstructed cervical spine structure (**C**: 1 week postoperatively, **D**: 5 years postoperatively), showing the fixation and integration of the implanted plate with the posterior lamina. CT, computed tomography.

After the surgery, the patient wore a Philadelphia collar for a month. X-ray and CT imaging of the cervical spine were performed again on the third day after removal of the drain. Anteroposterior and lateral radiographs suggested that the posterior cervical plates were well positioned and firmly fixed. CT scans of the sagittal spinal canal showed that the diameter was increased compared to before surgery (Figure 2C). Meanwhile, a three-dimensional reconstruction was performed to clearly visualize the posterior structure of the postoperative cervical spine, from which it was observed that the laminae from C3 to C7 were open and evenly fixed on both sides, rather than only on one side, as is traditionally seen (Figures 3A,B).

A week later, the patient’s neck pain had markedly diminished, the symptoms of weakness in both lower limbs were relieved, and the patient was able to walk with the help of walking aid. Although the patient retained some residual muscle strength in his right hand, physical examination revealed weakness in the right upper limb (power as evaluated by manual muscle testing: 4/5 for the deltoid muscles, biceps, and triceps), which was almost the same as before surgery. The total hospital stay in the conventional spine surgery department was 2 weeks, and the patient had virtually no neck pain, corresponding to a VAS score of 1, an mJOA score of 12, and an NDI score of 18, indicating moderate dysfunction.

Five years later, the patient returned to our hospital to undergo follow-up and complete tests. The patient was able to walk slowly on his own without the help of crutches, although handrails were needed to climb up and down the stairs. The weakness in his right upper limb had improved slightly, allowing him to use a spoon. Overall, the symptoms of spinal cord compression were relieved. The patient’s neck pain was completely absent, as expected. In addition, a series of x-rays showed that the position of internal fixation was well maintained, without significant loosening or displacement, and the cervical curvature and range of motion were the same as before (Figures 1E–H). Furthermore, axial CT scans of the operated cervical segments suggested a good fusion at the laminar hinge position, while the laminar doors were firmly kept open (Figure 2E), and the implanted plates were well fused with the posterior lamina, supported by the three-dimensional CT (Figures 3C,D). Finally, the MRI results revealed that the operated spinal canal had expanded sufficiently for the spinal cord to decompress adequately and that the operation had little effect on the symmetry of the posterior cervical muscles after 5 years (Figure 2D). According to a questionnaire administered to the patient, it was noted that the VAS score was 0, the mJOA score increased to 14, and the NDI score decreased to 12 already, indicating mild dysfunction. In summary, the patient experienced moderate improvement following surgery.

## 3. Discussion

Cervical laminoplasty, which ensures indirect posterior decompression by expanding the spinal canal to allow the spinal cord to migrate dorsally, is an effective method for patients with multiple disk herniations or OPLL (1). Although associated techniques have been continuously refined since its introduction, laminoplasty can be broadly categorized as unilateral open-door laminoplasty or double-door laminoplasty. Compared with the latter technique, which requires more surgical manipulations and one of which is performed directly on the midline of the compressed spinal cord, the former technique tends to be selected.

The original open-door laminoplasty expands the spinal canal by hinging the posterior arch on one side at the junction between the lamina and the lateral mass, while complete osteotomy is performed on the other side with greater compression and symptoms. The laminar door is kept open with the use of stay sutures that are placed through the spinous process and the facet capsule or the paravertebral muscle on the hinge side (9, 26). Later studies have described the use of suture anchors in the lateral mass for suture fixation and the use of translaminar screws to prevent door reclosure (23, 27). Although modified suture fixation techniques have substantially improved, surgeons began using more rigid fixation in the form of bone blocks and plates (Table 1) (11, 13, 28). Currently, plates are generally preferred because of their ease of application and the provision of immediate, stable fixation (29).

However, one of the criticisms of traditional open-door laminoplasty is the potential for increased axial neck pain (30). Ohnari et al. previously showed that the incidence of axial neck pain after laminectomy is 82.3%, which was significantly increased from the incidence of 59.1% before surgery (31). Furthermore, studies have suggested that axial symptoms may be caused by several problems, such as posterior extensor musculature intraoperative injury, destruction of the facet joints, and intraoperative nerve root damage (30, 32). Notably, during unilateral open-door laminoplasty, the canal is opened on one side and hinged on the other, which essentially creates an asymmetric expansion of the canal, further resulting in skeletal and muscular asymmetry that may result in postoperative axial pain and may further produce forces on the opened side, which can result in restenosis. Another issue with open-door laminoplasty is the lack of affordability for foraminal decompression at different sides due to its unilateral design. When radiculopathy exists simultaneously with different-sided foraminal stenosis, open-door laminoplasty may not be able sufficient to adequately relieve the patient's radicular symptoms well because of inadequate foraminal decompression or asymmetrical decompression.

Taking these challenges into consideration, our novel technique proposed the maintenance of spinal alignment, muscular symmetry, and motion force balance to reduce the associated pain. Distinct from the traditional procedure, construction of the hinges on the opposite sides of adjacent cervical segments meant that the laminar door could be opened alternatively from one end to the other in the cervical spine; thus, we termed this technique the alternating-side cervical laminoplasty. In the present study, we reported our experience with the case of a patient with cervical spondylotic myelopathy who agreed to undergo this novel procedure in 2017. Finite element (FE) analysis was performed before clinical surgery; it had validated that the postoperative structure after the novel technique employed would be beneficial in ensuring a balancing motion of the cervical spine. Furthermore, to ensure the stenosis of the neural foramen on different sides, this technique offered a flexible approach to choose an open side to decompress the neural foramen expediently as well as the canal. It could be expected that this technique would allow the intraoperative operation of multisite decompression to be simplified and the axial neck pain to be reduced. Optimistically, the strong postoperative recovery of the patient in our case also supports the use of this modification. Additionally, another alternative technique for reducing axial neck pain has been described, which involves the preservation of muscle attachment (33, 34). Riew et al. previously showed that surgeons should make every effort to preserve soft tissue on the C2 and C7 whenever possible as there appears to be a little downside to doing so, while reducing the incidence of postoperative neck pain (35). Therefore, we propose that the two modification techniques mentioned above can be applied simultaneously to minimize the occurrence of axial neck pain in the future.

## 4. Conclusion

Cervical laminoplasty represents a safe and effective posterior technique to achieve adequate exposure and decompression of the spinal canal required for the treatment of multilevel cervical spondylotic myelopathy. Here, we reported a case treated with a novel surgical technique for cervical laminoplasty, called the alternating-side cervical laminoplasty. This technique was proposed to maintain spinal alignment and muscular symmetry as much as possible by opening the laminar door alternatively, while simultaneously facilitating decompression of the neural foramen. Postoperative results after 5 years of follow-up showed that the patient experienced significant relief from his preoperative symptoms, with complete resolution of neck pain. Thus, besides preserving muscle attachment, the modified procedure mentioned above may be used to prevent postoperative axial pain. Certainly, a large number of clinical applications and postoperative results should be obtained to further evaluate its effectiveness before advocating it for popular implementation.

## Data Availability

The raw data supporting the conclusions of this article will be made available by the authors, without undue reservation.
